# Neoadjuvant intra-arterial chemotherapy in patients with primary lacrimal adenoid cystic carcinoma

**DOI:** 10.1186/1470-7330-14-19

**Published:** 2014-04-29

**Authors:** Sun Young Jang, Dong Joon Kim, Chang Yeom Kim, Cheng Zhe Wu, Jin Sook Yoon, Sang Yeul Lee

**Affiliations:** 1 Department of Ophthalmology, Soonchunhyang University Bucheon Hospital, Soonchunhyang University College of Medicine, Bucheon, Korea; 2 Department of Medicine, The Graduate School, Yonsei University, Seoul, Korea; 3 Department of Radiology, Yonsei University College of Medicine, Seoul, Korea; 4 Department of Ophthalmology, Severance Hospital, Institute of Vision Research, Yonsei University College of Medicine, Seoul, Korea; 5 Department of Ophthalmology, Yanbian University, Yanji, China

**Keywords:** Adenoid cystic carcinoma, Neoadjuvant intra-arterial chemotherapy, Complication

## Abstract

**Background:**

We describe four cases of primary lacrimal adenoid cystic carcinoma (ACC) treated with neoadjuvant intra-arterial chemotherapy (NAIC).

**Methods:**

The outcomes and complications of NAIC were reviewed. Several treatment-related local and/or systemic complications were noted.

**Results:**

One patient experienced ipsilateral eyelid and eyeball necrosis and permanent facial palsy; the second patient developed ipsilateral facial swelling and jaw claudication; and the third patient had febrile neutropenia, cellulitis, and phlebitis. These three patients underwent total exenteration. The fourth patient experienced neutropenia and thrombocytopenia and underwent tumour removal without exenteration. All patients were followed for more than 4 years and were alive at the last follow-up.

**Conclusion:**

Neoadjuvant intra-arterial chemotherapy may be an alternative treatment that will improve the survival rate of ACC. However, careful and close observation is needed to minimise the risk of side effects. Further investigations are needed to justify the use of chemotherapy-related treatments and the associated costs.

## Background

Primary lacrimal adenoid cystic carcinoma (ACC) is a rare, aggressive, malignant epithelial cancer with reported survival rates of less than 50% at 5 years and 20% at 10 years [1,2]. In our institution, the Severance Hospital, we usually perform total exenteration, neighbouring bone resection, adjuvant radiation therapy, and systemic chemotherapy in patients with ACC. Such aggressive treatments should be considered due to the poor prognosis of ACC [1,2]. Advances in imaging technology have enabled the detection of this cancer at an early stage. Moreover, supplementary treatments, such as radiation therapy and chemotherapy, also play an important role in enhancing survival rates by preventing recurrence and metastasis. In 1998, Meldrum et al. [3] first introduced the concept of neoadjuvant chemotherapy in the ophthalmological field through the treatment of two patients with advanced ACC of the lacrimal gland. The two patients had survived for 7.5 and 9.5 years at the last follow-up, thereby establishing the use of neoadjuvant chemotherapy in the treatment of ACC as a new paradigm. In 2006, Tse et al. [4] reported nine patients in whom neoadjuvant chemotherapy was used to treat ACC successfully, emphasising the usefulness of this treatment for local disease control and the enhancement of disease-free survival.

We performed neoadjuvant intra-arterial chemotherapy (NAIC) using the modified methods of Meldrum et al. [3] and Tse et al. [4] for four patients who visited our institution between February 2007 and May 2009. The aim of the present study was to report the complications related to NAIC. In addition, we also discuss an interventional method in terms of selecting an artery for NAIC that has not been reported previously in the literature.

## Methods

### Patients

This study was a non-comparative, consecutive case series. We conducted chart reviews of four survivors who visited the Severance Hospital, Yonsei University College of Medicine, Seoul, South Korea, between February 2007 and May 2009. All patients were diagnosed with primary lacrimal ACC and underwent NAIC. The patients had no intracranial infiltration or distant metastasis at the initial presentation. The tumour stage in all patients was T4N0M0 according to the staging criteria of the American Joint Committee on Cancer [5]. All patients were subjected to incisional biopsy to confirm the histological diagnosis. All patients agreed to the NAIC following full discussion of the poor prognosis of the disease, current treatment strategy, complications related to NAIC, and the uncertainty regarding the effectiveness of the treatment.

Demographics, clinical presentations, radiological imaging, and histopathological data were investigated. We reviewed the radiological images during the NAIC, the local and systemic complications after treatment, and the presence of local recurrence and distal metastasis. The status at the last follow-up was also evaluated. Approval to conduct this retrospective study was obtained from the Institutional Review Board of Severance Hospital of Yonsei University.

### Neoadjuvant intra-arterial chemotherapy technique

Bilateral internal and external carotid angiography was performed through a transfemoral approach with adequate precautions, using a 4 F or 5 F Headhunter diagnostic catheter (COOK MEDICAL INC., Bloomington, IN, USA). The primary feeding artery that stains the lacrimal gland (usually a branch of the lacrimal artery) was identified. The lacrimal artery can branch out from the ophthalmic artery (a branch of the internal carotid artery (ICA)) or from the middle meningeal artery (a branch of the external carotid artery (ECA)) as the meningo-lacrimal variant [6-8]. After defining the arterial anatomy, the diagnostic catheter was replaced for a 5 F Envoy® Guiding Catheter (Cordis Corporation, Miami Lakes, FL, USA). Subsequently, a Prowler® 14 microcatheter (Cordis Corporation) was coaxially introduced through the guiding catheter to attempt superselective cannulation of the artery feeding the lacrimal gland. The chemotherapeutic agent was infused through the microcatheter. During the chemoinfusion, intermittent fluoroscopic monitoring was done to ascertain adequate catheter position. We administered up to 3000 units of heparin intravenously during the procedure to prevent catheter-related thrombotic complications. Post-infusion angiography was performed to exclude any complications.

In our practice, NAIC needs 3 days of hospitalisation. On the first day of admission, intra-arterial chemoinfusion of cisplatin is done using the aforementioned technique; 60–80 mg/ m^2^ of cisplatin is diluted in 500 ml of normal saline solution and injected over 1 hour. Doxorubicin (20–25 mg/m^2^) is given intravenously for 3 days from the first day of hospitalisation. Thus, each chemotherapy cycle lasts 3 days, and the interval between two cycles is 21 days. A total of three cycles is regarded as completion of the regimen. Patients are followed up with computed tomography (CT) scans to assess the response. Orbital exenteration is done 3–4 weeks after the last NAIC cycle. Surgery is followed by radiation therapy (RT; dose, 6000 cGy) administered using a standard daily fraction protocol.

## Results

Between February 2007 and May 2009, a total of four patients with primary lacrimal ACC underwent NAIC. Patient demographics, outcomes, and the complications of NAIC are summarised in Table 1.

**Table 1:** Clinical summary of patients with lacrimal adenoid carcinoma treated with neoadjuvant intra-arterial chemotherapy

	**Age (years)/sex**	**Histological diagnosis**	**Location**	**NAIC**	**Surgery**	**Complications**	**Recurrence/metastasis**	**Outcome**
CASE 1	35/F	Cribriform-like ACC	Right orbit	2 cycles	Total exenteration	Ipsilateral eyelid and eyeball necrosis, permanent facial palsy	No	Survival
CASE 2	54/F	Tubular pattern ACC	Right orbit	3 cycles	Total exenteration	Facial swelling and tenderness, difficulties in chewing	No	Survival
CASE 3	45/F	Cribriform-like ACC	Right orbit	1 cycle	Total exenteration	Febrile neutropenia, cellulitis	No	Survival
CASE 4	75/M	ACC	Right orbit	3 cycles	Tumour resection only	Neutropenia, thrombocytopenia	Local recurrence	Survival

F, female; M, male; ACC, adenoid cystic carcinoma; NAIC, neoadjuvant intra-arterial chemotherapy.

### Patient 1

A 35-year-old pregnant woman presented with a 3-month history of progressive right proptosis. Exophthalmometry showed a 2-mm proptosis in the right eye. She was pregnant and hence denied any further investigations. At a later follow-up, 1 month from childbirth, a right-sided proptosis of approximately 4 mm was identified and it was accompanied by ocular pain and conjunctival hyperaemia. Her vision was 20/20 in both eyes, and her intraocular pressure was 17/15 mmHg.

**Figure 1:**
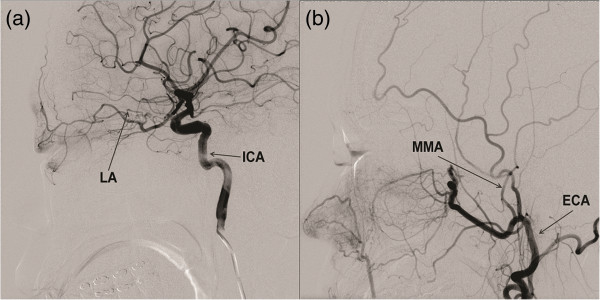
**Patient 1 (35-year-old woman). (a)** Lateral intracarotid angiogram shows the lacrimal artery (LA) originating from the ophthalmic artery. **(b)** The extra-carotid angiogram does not show tumour vascular staining or flow towards the orbit. ECA, external carotid artery; ICA, internal carotid artery; MMA, middle meningeal artery.

During NAIC, the ECA angiogram did not show tumour vascular staining or identified flow towards the orbit. The ICA angiogram showed the main blood flow supply to the orbit via the ophthalmic arterial branch (Figure 1). We performed NAIC through the ophthalmic artery after obtaining the patient’s consent because total exenteration after completing the NAIC had already been planned. The Prowler 14 microcatheter was positioned at the proximal end of the ophthalmic artery, which supplied the orbit, including the lacrimal grand. Contrast injections through the microcatheter did not show significant reflux into the cerebral circulation. Cisplatin (80 mg/m^2^) was infused with no complications. Systemic doxorubicin (25 mg/m^2^) was administered intravenously.

**Figure 2:**
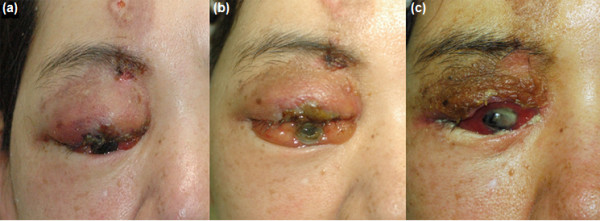
**Patient 1 (35-year-old woman).** Eyelid and eyeball necrosis as local complications of intra-arterial chemotherapy, resulting from the selection of the internal carotid artery. Photograph was taken 1 week **(a)**, 2 weeks **(b)**, and 1 month **(c)** after neoadjuvant intra-arterial chemotherapy.

One week after NAIC, necrosis of the right upper eyelid was noted, and the patient complained of hypoaesthesia on the forehead. Gradually, the entire eyelid necrosed and the eyeball was exposed (Figure 2). Systemic complications, such as gingivitis, oral ulceration, alopecia, general weakness, and neutropenia (absolute neutrophil count of < 1500) were noted. The second NAIC angiogram, which was obtained after 6 weeks, showed hypotrophy of the right ophthalmic artery, a small collateral flow towards the orbit from the middle meningeal artery (MMA), and a distal maxillary artery from the ECA. The microcatheter was positioned in the distal portion of the main ECA trunk, and cisplatin was injected.

Four days later, right facial palsy was observed. Because of the local and systemic complications, the third cycle of NAIC was cancelled. Total exenteration was performed, and RT (6000 cGy) was performed 3 months later. Right facial palsy remained after the total exenteration. At the 69-month follow-up, the patient was disease-free.

### Patient 2

A 54-year-old woman presented with a 1-month history of right eye vision loss with progressive proptosis. Her visual acuity at the first visit was 20/400 in the right eye and 20/20 in the left eye. Her intraocular pressure was 19/19 mmHg. Right proptosis of 6 mm was noted.

A CT scan showed a solid tissue mass with an irregular margin in the right orbit, with direct invasion into the adjacent bony orbit. The tubular pattern typical of ACC was confirmed on incisional biopsy.

**Figure 3:**
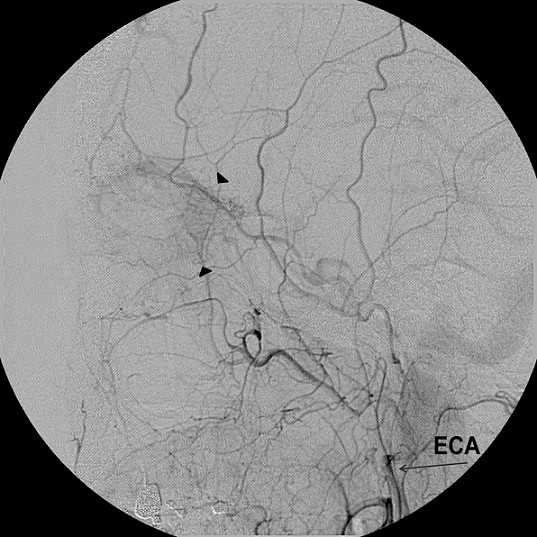
**Patient 2 (54-year-old woman).** Tumour staining (arrowheads) was seen primarily from the lacrimal branch of the right middle meningeal artery, which originated from the external carotid artery (ECA). This staining enabled selection of an artery from the ECA branches.

**Figure 4:**
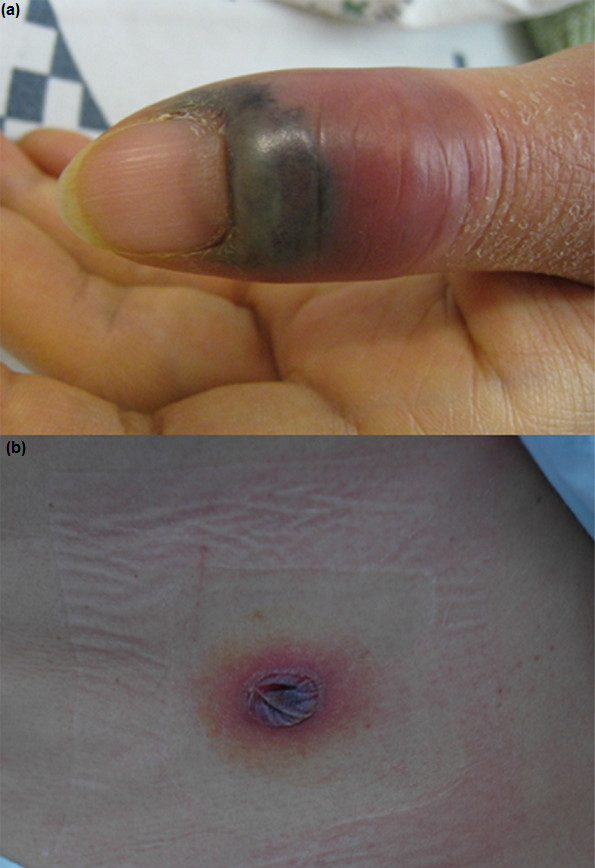
**Patient 2 (54-year-old woman). (a)**: Cellulitis of the thumb. **(b)** Viral blister on the abdomen due to neutropenia as a systemic complication.

At angiography, the tumoural staining was seen during the ECA injection (Figure 3). The microcatheter was inserted into the distal portion of the main trunk of the ECA, just proximal to the MMA, and cisplatin (80 mg/m^2^) was infused for 1 hour. Doxorubicin (25 mg/m^2^) was injected intravenously. Five days after NAIC, the left thumb of the patient was pricked by a thorn; she subsequently developed pain, swelling, fever, and general weakness. Her body temperature was 39°C, and her absolute neutrophil count was 530. The patient was diagnosed with febrile neutropenia, cellulitis, and phlebitis and required hospitalisation (Figure 4a). Erythematous blisters of various sizes were found on her forehead, abdomen, and tibia (Figure 4b). On consultation with the Dermatology Department, the blisters were determined to be viral in origin. Aciclovir cream was applied, and the lesions were controlled.

Although viral infection during the immunosuppression phase of chemotherapy is not inherent to the intra-arterial route of delivery [9], we reduced the dose of cisplatin from 80 to 60 mg/m^2^ due to the systemic complication. The dose of doxorubicin was also reduced from 80 to 60 mg/m^2^ when the second and third NAIC cycles were performed. All three cycles of the NAIC regimen were completed in this patient, as originally planned.

Approximately 5 weeks later, total exenteration was performed. At the same time, bony erosion of the superior and lateral orbital rim was noted, and adjacent bone was removed using a rongeur. RT was initiated 6 weeks after surgery. No local recurrence or distal metastasis was observed over the next 59 months.

### Patient 3

A 45-year-old woman presented with a 1-year history of eyelid swelling. The discomfort and lid oedema had aggravated over the last 3 months. At the time of presentation, visual acuity was 20/25 in both eyes, and the intraocular pressure was 16/16 mmHg.

**Figure 5:**
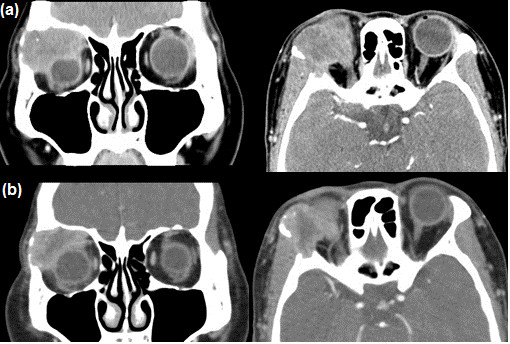
**Patient 3 (45-year-old woman). (a)**: Computed tomography (CT) scan taken before neoadjuvant intra-arterial chemotherapy (NAIC) and **(b)** CT scan after NAIC. Note the reduction in tumour size.

On physical examination, a hard, immovable mass was palpable in the right superotemporal area. A CT scan showed an ill-defined, soft tissue mass (Figure 5a). At the incisional biopsy, an ACC of cribriform type was confirmed and NAIC was planned. Cisplatin (80 mg/m^2^) was injected into the internal maxillary artery proximal to the MMA. Doxorubicin (25 mg/m^2^) was administered intravenously.

Three days after NAIC, the patient complained of right facial pain, swelling, and discomfort on opening the mouth. Erythema was noted on the right cheek and neck. We infused dexamethasone (10 mg) intravenously, and an ice bag was applied. The erythema resolved. A follow-up CT obtained after 3 weeks showed that the size of the tumour had decreased from 3 to 2.3 cm (Figure 5b). However, the patient did not want to continue the NAIC; therefore, we could not complete the full NAIC regimen. After 1 month, total exenteration of the right orbit was performed. Removal of the lateral zygomatic bone was performed because of cancer invasion of the lateral orbital rim. In accordance with the therapeutic plan, postoperative irradiation (6000 cGy) was performed. We followed the patient for 48 months. At the last visit, the right inferior orbital rim showed increased intensity on the magnetic resonance imaging scan, and the patient is currently being followed up closely.

### Patient 4

A 75-year-old man presented with a 3-month history of right eye pain and proptosis. He was diagnosed with ACC, and total exenteration was recommended at another hospital. He did not consent to this radical treatment. At the first visit to our hospital, his visual acuity was 20/25 in the right eye and 20/20 in the left eye. His intraocular pressure was 21/18 mmHg, and a 5-mm right eye proptosis was noted.

**Figure 6:**
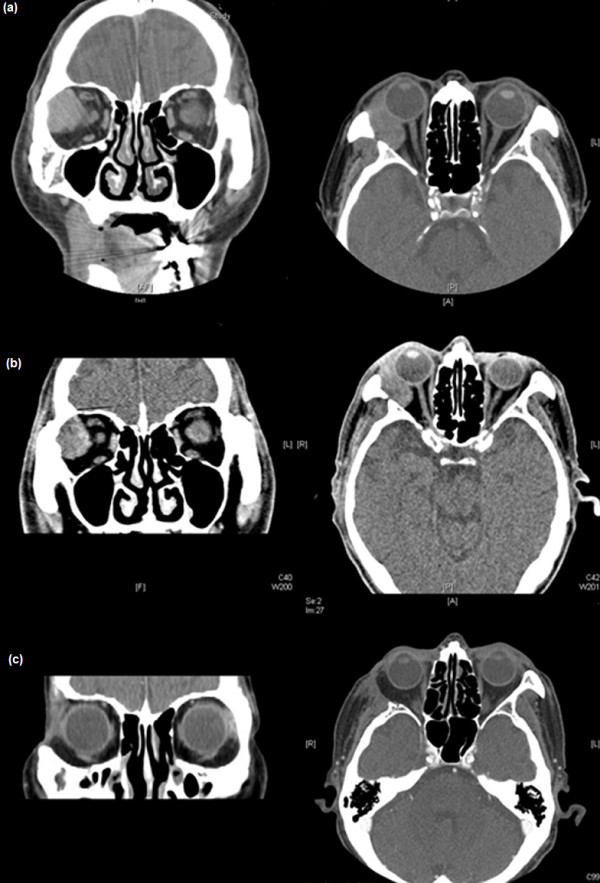
**Patient 4 (75-year-old man). (a)** Preoperative computed tomography (CT) scan. **(b)** CT scan obtained after neoadjuvant intra-arterial chemotherapy (NAIC). Note the reduction in tumour size after NAIC. **(c)** CT scan obtained after tumour resection with adjacent bone removal.

A CT scan (Figure 6a) showed a soft tissue mass in the superolateral right orbit. NAIC was performed as discussed earlier. An angiogram showed 56% vascular stenosis of the ICA. We selected the distal main trunk of the ECA just proximal to the MMA for NAIC, and cisplatin (80 mg/m^2^) and systemic doxorubicin (25 mg/m^2^) were administered.

All three cycles of the NAIC regimen were completed, and definite size reduction was observed after the treatment (Figure 6b). Specific local complications related to NAIC were not encountered, and mild decreases in white blood cell and platelet counts were observed after the second NAIC cycle.

After completion of the NAIC regimen, the patient underwent mass removal with adjacent bone resection (Figure 6c). External RT (6300 cGy) was performed 1 month after surgery. His visual acuity during the follow-up period was 20/30 in the right eye and 20/20 in the left eye. Proptosis was not observed on performing exophthalmometry.

After 54 months from treatment, there was local recurrence of the tumour for which exenteration was recommended.

## Discussion

Tse et al. [4] recently reported that the cumulative 5-year ACC-specific death rate was 57.1% in a conventional treatment group, but only 16.7% in an intra-arterial chemotherapy treatment group. The cumulative 5-year recurrence rate was 71.4% in the conventional group and 23.8% in the chemotherapy group, emphasising the usefulness of intra-arterial chemotherapy in local disease control and in improving overall disease-free survival. Because ACC is extremely rare and has a poor prognosis, these results raise the possibility of using intra-arterial chemotherapy in the treatment of this disease. However, to date, few reports detailing the use of intra-arterial chemotherapy for ACC have been published [3,4]. The effectiveness of most therapies for ACC is judged on the basis of retrospective case reports or on expert opinion because of the rarity of the condition; therefore, complications related to introducing new therapeutic modalities are worth noting. We have thus reported the complications that we experienced during intra-arterial chemotherapy and have further discussed interventional methods resulting in local complications.

With respect to selecting an artery for chemotherapy, Meldrum et al. [3] and Tse et al. [4] recommended using the ECA to inject a chemotherapeutic agent for treatment of lacrimal gland tumours. The authors explained that the ECA was the preferred artery because of concerns that the chemotherapeutic agents could be delivered into the cerebral circulation when injecting into arterial feeders originating from the ICA [4]. However, in our experience, the feeding vessel to the tumour may not be always obvious on the ECA angiogram. Patient 1 had main lacrimal feeders from the ophthalmic artery of the internal carotid system instead of the ECA. Because of this anatomical disposition and because exenteration of the eye had already been planned, and further because contrast injections through the microcatheter did not show reflux into the cerebral circulation, we opted to infuse through the ICA feeder. Understanding the anatomical variations of the blood vessels that supply the lacrimal gland is required prior to performing intra-arterial selection. The lacrimal artery can branch out from the ophthalmic artery originating from the ICA, but it can also branch out from the MMA originating from the ECA. Moreover, it can receive a blood supply from both [6-8]. The orbit can receive a collateral supply from the distal maxillary artery and the infraorbital, sphenopalatine, and anterior deep temporal arteries. Because the ophthalmic artery was selected in patient 1, necrosis occurred in the eyeball as well as in the eyelid that received the blood supply from this branch. The toxicity of the antitumour agent is suggested as the cause of the eyelid and eyeball necrosis in this case. Although in this case there were no complications related to the chemotherapeutic agent being delivered into the cerebral circulation, the risk of injection into the cerebral circulation when selecting the ICA should be kept in mind.

In the present study, all patients survived until the last follow-up. These outcomes are in accordance with the successful results pertaining to local disease control and overall disease-free survival that have been reported previously [4]. Imaging technology and supplementary treatments, including intra-arterial chemotherapy, are expected to play a positive role not only in enhancing survival rates, but also in preventing orbital exenteration. Several recent reports have stated that good survival is achieved by removing only the tumour and using plaque radiotherapy, without performing exenteration [10,11]. These authors reported that conservative treatment was possible because radiation could be used for residual cancer cells and that this treatment would be especially useful for small children and monocular patients. However, in the present study, the patient who underwent only tumour resection and not exenteration (patient 4) showed tumour recurrence at the last follow-up. The tumour cells can exit the orbit along either the lacrimal nerve or the blood vessel from the time of diagnosis because of the micrometastasis and perineural invasion characteristics of ACC. To conclude that intra-arterial chemotherapy might prevent this type of local and possibly metastatic aggressiveness, further study is needed.

## Conclusion

In conclusion, NAIC may increase the survival rate of patients with ACC and may represent a new treatment option for preservation of the eye in such cases. However, to decrease treatment-related complications and ensure completion of the three-cycle protocol, close observation through proper follow-up after treatment and appropriate management of adverse effects are necessary.

## Competing interest

The authors declare that they have no competing interests.

## Authors’ contributions

We would like to thank all of the following: (SYJ, DJK, SYL) for the design and performance of the study; (SYJ, DJK, JSY, CYK, CZW) for the collection of data; (DJK, JSY, SYL) for the management, analysis, and interpretation of data; (SYJ, SYL) for the preparation of the manuscript; and (DJK, SYL) for the review and approval of the manuscript. All authors read and approved the final manuscript.
